# Characterizing Human Box-Lifting Behavior Using Wearable Inertial Motion Sensors

**DOI:** 10.3390/s20082323

**Published:** 2020-04-18

**Authors:** Steven D. Hlucny, Domen Novak

**Affiliations:** Department of Electrical and Computer Engineering, University of Wyoming, Laramie, WY 82071, USA; shlucny@uwyo.edu

**Keywords:** IMU, lift, characterization, classification, wearable sensors, human motion, analysis

## Abstract

Although several studies have used wearable sensors to analyze human lifting, this has generally only been done in a limited manner. In this proof-of-concept study, we investigate multiple aspects of offline lift characterization using wearable inertial measurement sensors: detecting the start and end of the lift and classifying the vertical movement of the object, the posture used, the weight of the object, and the asymmetry involved. In addition, the lift duration, horizontal distance from the lifter to the object, the vertical displacement of the object, and the asymmetric angle are computed as lift parameters. Twenty-four healthy participants performed two repetitions of 30 different main lifts each while wearing a commercial inertial measurement system. The data from these trials were used to develop, train, and evaluate the lift characterization algorithms presented. The lift detection algorithm had a start time error of 0.10 s ± 0.21 s and an end time error of 0.36 s ± 0.27 s across all 1489 lift trials with no missed lifts. For posture, asymmetry, vertical movement, and weight, our classifiers achieved accuracies of 96.8%, 98.3%, 97.3%, and 64.2%, respectively, for automatically detected lifts. The vertical height and displacement estimates were, on average, within 25 cm of the reference values. The horizontal distances measured for some lifts were quite different than expected (up to 14.5 cm), but were very consistent. Estimated asymmetry angles were similarly precise. In the future, these proof-of-concept offline algorithms can be expanded and improved to work in real-time. This would enable their use in applications such as real-time health monitoring and feedback for assistive devices.

## 1. Introduction

Musculoskeletal disorders caused by frequent or high-risk lifting tasks are among the most common work-related injuries among physical laborers worldwide, especially those in the fields of construction and factory assembly lines [1]. Methods exist for assessing the risk associated with lifts. For example, the National Institute for Occupational Safety and Health (NIOSH) designed the revised NIOSH lifting equation (RNLE), which allows users to compute a lifting index for any lift using several parameters [2]. The lifting index indicates the amount of “risk” associated with a lift such that lifts with a higher lifting index have a higher likelihood of causing injury. Parameters required to compute the lifting index include the weight of the object, the vertical height above the floor, the horizontal distance from the person, etc. Such methods can be great tools for engineering safer lifting tasks, but they still rely on subjective observations and do not account for human variability. Thus, a system for automatically monitoring lifting behavior over time could prove useful. Furthermore, the information provided by such a real-time system could be used as control feedback for assistive devices such as trunk exoskeletons that support the user during lifting tasks, thereby preventing musculoskeletal disorders or reducing the consequences of such disorders [3,4,5].

For human motion analysis problems such as this one, optical tracker-based systems such as Vicon (Vicon Motion Systems Ltd., Oxford, UK) or Optotrak (Northern Digital Inc, Ontario, Canada) are often used [6]. These systems rely on multiple fixed cameras in a room and physical markers placed on the subjects’ bodies to produce measurements. They can be very accurate in a controlled laboratory setting, but are not very adaptable to different work environments due to a lack of portability, and because they are affected by physical obstructions, limited field of view, and adverse lighting conditions [6,7]. It is unlikely that enough markers can be used to capture the full 3D pose using just vision processing. A common technique is to create a skeletal model of a human, then perform inverse kinematic analysis on the known marker positions (captured with the computer vision system) to move the joints in a realistic way. One defining characteristic of optical systems is that their measurements are absolute; their accuracy is limited only by what the cameras can resolve and the fidelity of the human inverse kinematic analysis that is done in software. This characteristic makes them a popular choice as references for other motion capture techniques. The biggest weakness of optical systems, like force plates and other fixed measuring devices, is that they are not portable enough for field use.

Wearable sensors are likely the key to achieving reliable motion analysis in realistic environments. They can be fully self-contained on the wearer’s person so that they can move freely during the analysis, and on-body recording makes moving large distances in extreme environments possible while recording. Furthermore, wearable sensors have already been used for a wide variety of motion analysis applications. Several studies have focused on human gait analysis using wearable inertial measurement units (IMUs—devices consisting of accelerometers, gyroscopes, and optionally magnetometers) for both normal, [8,9,10], and abnormal gaits [11,12]. Gait measurements captured with IMUs can also be used as input to lower limb assistive devices, such as robotic prostheses and exoskeletons [13]. They can be used to detect dangerous conditions, such as falling in geriatric populations [14]. Wearable sensors have also been used extensively for kinematic analysis of the upper limbs [15]. For example, it is possible to assess the progress of arm rehabilitation [16] and to monitor the effectiveness of shoulder surgery [17]. Due to their form factor and cost-effectiveness, they can also be used as an input for rehabilitation games [18]. Recently, there has been increasing interest in using wearable sensors for analyzing human motion in athletics, such as swimming strokes [19] and the kicking of footballs [20]. This is only viable because of the portability and cost-effectiveness of IMUs.

IMU technology has advanced to the point where full-body motion capture is now possible with applications in many fields [21,22]. The 3D linear acceleration measured by the accelerometers, 3D angular rate measured by the gyroscopes, and 3D magnetic field measured by the magnetometers can be fused together with a sensor fusion algorithm (commonly a Kalman filter) to produce the absolute orientation of the device in 3D space. In crude implementations, the angular rates from the gyroscopes can simply be integrated over time to compute the orientation of the device. However, the measurements will inevitably drift due to accumulations of error caused by noise, temperature bias, or sensor error, which means that the device will require constant recalibration. Sensor fusion reduces the severity of this issue by adjusting the coordinate frame in reference to acceleration due to gravity (measured by the accelerometers) and the Earth’s magnetic field (measured by the magnetometers). When multiple IMUs are attached to various segments of the body, the orientations of those segments can be measured. Using inverse kinematics with a skeletal model of the human body, it is possible to measure joint angles, segment positions, velocities, etc. to produce a full-body pose reconstruction.

Some studies have been done on using wearable sensors to analyze human lifting behavior. For instance, Brandt et al. proposed a method of identifying a lift as low or high risk using data from two accelerometers placed on the back and surface electromyography (sEMG) electrodes on the upper trapezius and erector spinae muscles [23]. They designed a set of lifts for the participants to perform and only varied the load between trials. They showed that it is possible to estimate the load being carried for a known type of lift, and then use that to classify the lift as low or high risk, for which they achieved accuracy as high as 78.1% using a subject-specific threshold based on sEMG and back inclination (obtained from the accelerometer). However, the starts/ends of the lifts were not automatically detected and the posture (e.g., stooping or squatting) used throughout the lift, the location of the object, and the amount of twisting involved were not measured.

Lu et al. [24] developed an algorithm that used IMUs to measure lifting risk factors in real time. Five IMUs were attached to specific locations on the subjects’ bodies and the data was fed into two separate software modules: a lift detection module and a sensor fusion module. The lift detection module monitored the IMU data in 2.5 s sliding windows with a 0.5 s step size and used a machine learning approach to determine whether the wrist sensors were “synchronized,” meaning that the hands were inertially coupled. An assumption was made that when the wrists were synchronized, a lift was most likely occurring. The authors manually labeled every 2.5 s window in 25 min of training data, then compared it to the results of the lift detection module. The module correctly labeled 83%–85% of the actual lift windows as lift windows (true positives), and it mislabeled 32% of the non-lift windows as lift windows (false positives). The sensor fusion module processed the output from the IMUs’ accelerometers and gyroscopes to produce absolute orientations in real time. These orientations were used to calculate the trunk flexion angle, the vertical height of the object, and the horizontal distance from the object to the lifter. In a second related study [25], the authors compared the estimated values to values measured by a commercial motion capture system and found that estimation of the vertical and horizontal positions of the box were poor with mean errors of 33 cm and 6.5 cm, respectively. The estimate of trunk flexion had a mean error of 2.3 degrees. However, these studies have several weaknesses: the features used for lift detection are unclear, and the authors do not report what percentage of the windows in the training data included true synchronization, so the actual training accuracy cannot be calculated. Lift detection accuracy is discussed in the second paper, but they do not mention whether any of the lifts were completely missed by the algorithm. Furthermore, the approach does not consider the asymmetry of the lift (in this study, asymmetry is defined as the twisting of the upper body relative to the lower body required to complete the lift) or the weight of the object, both of which are important factors for determining the risk associated with a lift with the NIOSH lifting equation [2]. The authors also did not attempt to classify the posture used throughout the lift, which would be useful for health monitoring or assistive device control.

O’Reily et al. [26] discuss a method of classifying deadlifts (a popular weight-lifting exercise for rehabilitation and strength training) as good or bad using wearable IMUs. Two experiments were carried out: one in which the participants deliberately performed aberrant deadlifts mixed with acceptable ones, and one in which they performed a 3-repetition maximum strength deadlift protocol to elicit aberrant form naturally. The authors defined five categories of deadlifts for the purpose of classification: acceptable, shoulders behind bar at start position, rounded back at any point during movement, hyperextended spine at any point during movement, bar tilting, and other. Random forest classifiers were trained with 17 descriptive features to perform both binary classification (acceptable or aberrant) and 5-category classification. Using five sensors placed on the lower back, left and right thighs, and both shanks, they were able to achieve 93% cross-validation accuracy in binary classification with personalized classifiers and 75% with a global classifier for the lifts from experiment 1. The multi-class personalized classifiers achieved 81% accuracy, whereas the global classifier achieved 60%. For experiment 2, the personalized classifiers were 84% and 78% accurate for binary and multi-class, respectively, while the global classifiers were 73% and 54% accurate. A characterization like this is similar to what we would like to achieve for general lifts. However, it is necessary to develop a lift detection algorithm to temporally locate the beginning and ending of the lifts. Classification-only algorithms are also potentially limited in their ability to characterize a wide range of lift types (it is difficult to design categories for every possible case), so it is important to take other continuous measurements, such as vertical height of the object, asymmetry angle, etc.

Most of these studies are too narrow in scope to be used to characterize general lifting behavior. Furthermore, they do not focus on classifying important features such as posture and asymmetry. They are also restricted by the amount of information they can gather from a limited number of sensors. Therefore, there is a need for further research. IMUs were the sensor of choice for this study, as they are simple, cost-effective, noninvasive, and highly portable compared to alternative motion tracking solutions [10]. The primary objective of this study was to develop an offline pattern recognition algorithm that can detect and characterize human lifting activity. Ideally, the algorithm would be able to extract information on the posture used throughout the lift and the approximate distance the object was moved. Information about the object being lifted, such as weight, would also be useful. However, these are difficult to get from IMU data alone as IMUs provide no way to measure them directly. For this study, we assumed that a lift can be broken down into the following components.

1. Source: The starting location of the object relative to the lifter.
2. Destination: The final location of the object relative to the lifter.
3. Asymmetry: The amount of twisting required to perform the lift.
4. Posture: For the purposes of this study, whether the lifter was squatting or stooping during the lift.
5. Weight: The estimated weight of the object being lifted.

This information includes the parameters necessary for computing the NIOSH lifting index [2] and could serve as input to an assistive device’s control algorithm.

## 2. Methods

### 2.1. Hardware

**Figure 1:**
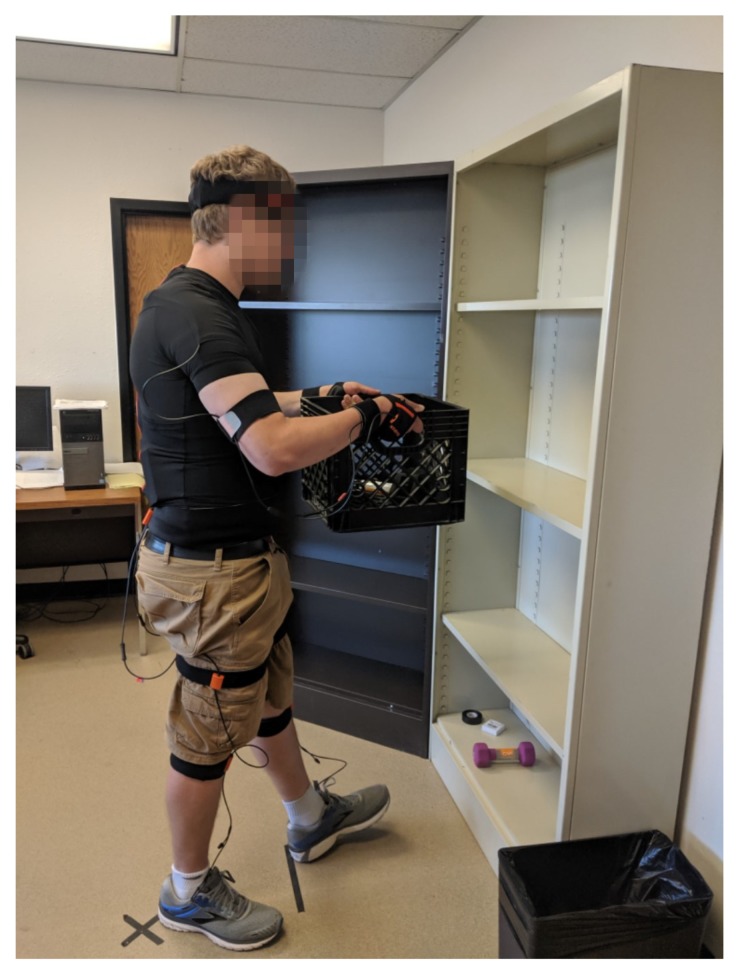
Participant wearing the Xsens Link system while performing a lift.

A commercial IMU system was used to obtain motion measurements and joint angles: Xsens Link (Xsens Technologies BV, Enschede, Netherlands). Link is a full-body motion capture system consisting of 17 IMU “trackers” attached to the feet, lower legs, upper legs, pelvis, shoulders, sternum, head, upper arms, forearms, and hands. Each sensor contains 3D linear accelerometers, 3D rate gyroscopes, 3D magnetometers, and a barometer [27]. Full specifications can be found in the MVN User Manual [27], but the basic tracker performance is characterized as follows; static accuracies for roll/pitch and heading are 0.2 and 0.5 degrees, respectively; dynamic accuracy is 1 degree (root mean square); accelerometer range is ±16 g; and gyroscope range is $\pm 2000^{\circ }$/s. They are attached to the body using a tight-fitting pocketed shirt and hook-and-loop straps, and measure the motions of each body segment. Table 1 describes the positions of the sensors on the body and a participant wearing the system is shown in Figure 1.

3D tracking data from each of the motion trackers are transmitted to a workstation computer wirelessly at 240 Hz. There, the Xsens MVN Fusion Engine combines the data from the individual motion trackers with a biomechanical model of the subject’s body to obtain segment positions and orientations [21]. A calibration routine was carried out for each participant to account for sensor position/orientation and body shape variance. In this routine, the participants’ dimensions (body height, foot length, arm span, ankle height, hip height, hip width, knee height, shoulder width, shoulder height, and shoe sole height) were first measured and entered into the software. Next, they were asked to hold a specific static pose for several seconds, then walk back and forth about 10 feet. The Fusion Engine determined the orientation and position of the sensors relative to their segments during this process. The MVN biomechanical human model has 23 segments with 22 joints. Each joint is specified by statistical parameters for 6-degree-of-freedom joint laxity and an advanced model is used to solve the kinematics of the spine and shoulder blades. The output from the Fusion Engine is a full kinematic description of each segment, which includes position, velocity, acceleration, orientation, angular velocity, and angular acceleration. Thus, the Xsens motion capture system enables full-body pose reconstruction comparable to optical systems, with about a 1% error in segment traveled distance without additional fusion with optical systems, GPS, etc. Although the system records at 240 Hz, the data were downsampled to 60 Hz for analysis.

### 2.2. Experimental Design

An experiment was carried out in which participants performed lifts while wearing the IMUs. Twenty-four volunteer participants (19 male, 5 female, 30 ± 9 years old, 176.6 ± 8.6 cm tall, 79.9 ± 18.6 kg weight) were recruited from the students and staff of the University of Wyoming. Exclusion criteria excluded people with conditions that may affect their ability to perform lifts safely or normally. These criteria included a history of major spinal injuries or surgery (e.g., disc removal, spinal fusion, and hardware placement), chronic or current acute lower back pain, and pregnancy. Each participant signed an informed consent form prior to beginning the experiment and the study protocol was approved by the University of Wyoming Institutional Review Board (protocol #20190801SH02476). Participants were asked to wear closed-toe shoes suitable for exercise and to avoid very loose-fitting clothing that would interfere with the sensor straps.

Adjustable shelving was used to provide source/destination locations at the knee (54 cm), chest (108 cm), and head (157 cm) levels. These levels were fixed across all experiments and did not necessarily align with the knees, chest, and head of every participant. The floor was also used as a location, for a total of 4 locations. The 2 shelves were situated 45 degrees apart from each other, as shown in Figure 1.

Each participant performed a pre-determined set of lifts consisting of 30 main lifts and 1–3 transition lifts. The order of the main lifts was randomized for each experiment to avoid the effects of muscle fatigue on the overall data trends. As a consequence, the crate sometimes needed to be moved from one location to another between main lifts. Rather than just telling participants to move it, “transition” lifts were included in the predetermined set of lifts and were recorded to obtain additional data. These lifts were sometimes members of the main lift group, and were other times specifically designed for the transition. Lifts that were included for transitions, but were not main lifts, were called “extra” lifts. For data integrity, each main lift was performed 2 times, for a total of 60 main lifts plus 1–3 transition lifts per experiment. Mistakes during the trials (either by the participants or by the experimenter) and some bad recordings caused some lifts to be performed more or fewer times than planned. In 4 instances, unplanned lifts were also mistakenly performed. Because these lifts were not deemed harmful to the study, they were kept in the data for analysis. Unless otherwise stated, all lifting trials (main, extra, and unplanned) were processed by the algorithms. Table 2 summarizes the planned and actual occurrences of each lift.

Participants were given at least 15 s of rest between lifts. The main object was a plastic crate (33 cm wide, 33 cm long, and 28 cm tall) with handles that weighs 10.4 kg. The secondary object was a similar crate that weighed 3.6 kg. For the remainder of this paper, the primary object will be referred to as the 10 kg weight, and the secondary object the 3 kg weight.

**Figure 2:**
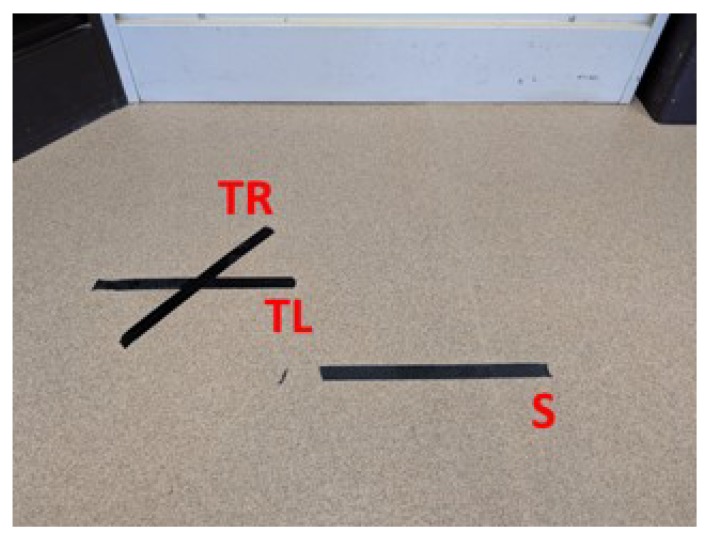
Tape lines on the floor for start/end crate alignment. The tape line marked S is the start/end point for straight lifts and was parallel to and 56 cm away from the main shelf (white). TL was the starting point for lifts in which the participant twisted left while lifting from the floor to the shelf and was parallel to and 42 cm away from the main shelf. TR was the starting point for lifts in which the participant twisted right while lifting from the floor to the shelf and was parallel to and 42 cm away from the secondary shelf (brown).

To ensure consistency across subjects, tape marks on the floor indicated where the crate should be placed for the lifts that start or end on the floor level. The marks were arranged in front of the shelves, as shown in Figure 2.

**Figure 3:**
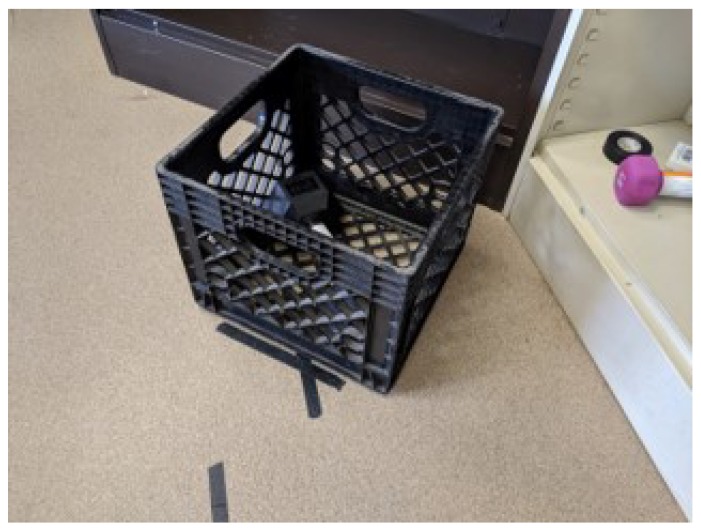
Crate aligned with a tape mark at the beginning of a twisting lift.

When the floor was the starting point, the crate was initially placed in front of the appropriate line, like the example in Figure 3. Likewise, participants were instructed to place the crate in front of the appropriate line when the floor was the destination.

Table 2 shows the full list of lifts performed by each participant.

For the asymmetric lifts, the twist direction indicates which way the participant twisted while carrying the object. The participants always started facing toward the lower of the source/destination pairs. For example, for the “floor to chest, squatting, 10 kg, twisting left” lift, participants were instructed to start facing the object on the floor. Once the object was picked up, the participants twisted to the left to place it on the shelf. For the “chest to floor, squatting, 10 kg, twisting right” lift, participants started facing the location on the floor where the object would eventually be placed. They then twisted left, grabbed the object from the shelf, twisted back to the right, and placed it on the floor.

Participants were instructed to perform lifts with either the ergonomically correct posture (squatting) or with incorrect posture (stooping) [28]. In squatting lifts, participants attempted to keep their backs straight while doing most of the lifting with their legs. In stooping lifts, they bent over to pick up the object while keeping their knees mostly straight. Lifts from chest to head and vice versa do not require any particular posture.

### 2.3. Lift Detection

**Figure 4:**
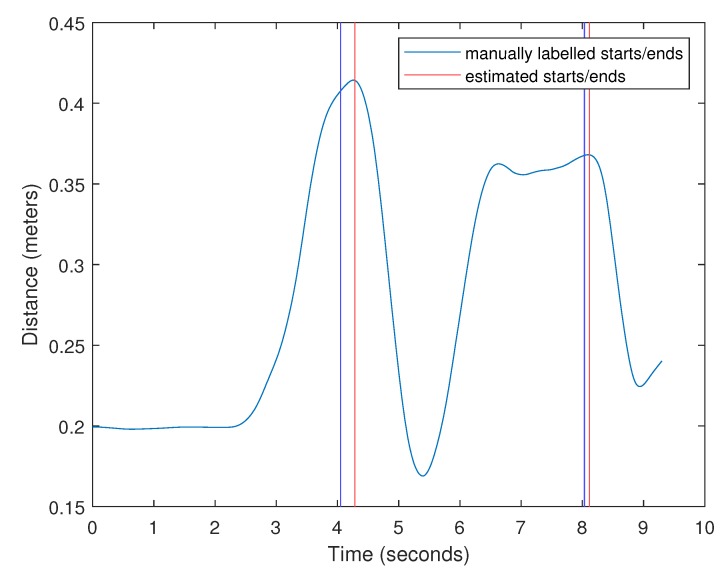
Lift start/end detection using the distance from the center of mass to the hands.

The first step to characterizing a lift is identifying its starting and ending time. This allows us to determine the starting and ending location of the object as well as the duration of the lift. The data were pre-segmented into individual lifting trials plus some time before/after the lift during the experiment. Only one lift was performed per trial, and the participants’ hands always started from the same position (relaxed, hanging by their sides). We hypothesized that, at the beginning and ending of the lift, the participant’s hands would be the furthest away from their center of mass. We made this assumption on the basis that human balance reactions cause the center of mass to remain vertically aligned with the base of support (in this case, the feet) [29]. As the arms are extended away from the body, the distance from the hands to the center of mass increases. The MVN Analyze software produces the participant’s estimated center of mass and hand positions over time. The lift detection algorithm computes the mean distance from the center of mass to the participant’s left and right hands for every frame of the trial. A moving-average filter with a window size of 30 points is applied, and then the two most prominent peaks in the trial are identified as the beginning and ending of the lift. The prominence of a peak measures how much it stands out due to its intrinsic height and its location relative to other local maxima. To enable comparison, the start/end times were manually labeled for each trial. The first author did this by watching the visual lift playbacks (with MVN Analyze), and estimating the times when the majority of the crate’s weight was transferred from its supporting platform (shelf or floor) to the participant and vice versa. Figure 4 shows a plot of the distance computed by the lift detection algorithm along with the estimated and manually labeled start/end times.

### 2.4. Parameter Estimation

Certain lift parameters may be computed directly from the IMU data without using machine learning techniques. Algorithms were implemented to calculate the asymmetry angle, the vertical height of the object above the floor, the vertical displacement, and the horizontal distance between the object and the midpoint between the person’s feet. These parameters were computed using both the manually and automatically labeled start/end times.

Lift asymmetry is defined as the amount of “twisting” of the trunk required to complete a lift. To calculate this angle, the program identifies two unit vectors on the floor plane: the reference vector and the maximum twist vector. The reference vector begins at the midpoint between the participant’s feet and extends forward in the direction the person is facing. The maximum twist vector originates from the same location, but extends in the direction of the hands at the beginning or end of the lift, depending on which involves more twist. The angle between these vectors is calculated as the asymmetry of the lift. The locations of the hands and feet are obtained from the MVN Analyze software, and the direction the participant is facing is determined by finding the mean orientation of the feet. The twist amount was not precisely controlled during the experiment. The shelves were situated 45 degrees apart from each other, but participants were free to twist as much or as little as they needed. Due to the placement of the shelves, we expected the mean twist angles over all the twisting lifts to be approximately 45 degrees.

The vertical height of the object above the floor at the beginning and ending of the lift can be estimated using the position of the hands. If we assume that the midpoint between the hands is the location of the object being carried, then the vertical height can easily be computed using the estimated height of the hands. The program extracts the location of the hands at the beginning and ending of the lift from the IMU data, then calculates the midpoint between them to estimate the location of the object. The shelf heights were fixed across all participants, so the vertical heights of the lift sources and destinations are known. To calculate reference heights, 30 cm (the approximate height of the hands relative to the bottom of the crate) was added to each of the shelf heights provided in Section 2.2. Thus, the reference heights are 30 cm for floor, 84 cm for knee, 138 cm for chest, and 187 cm for head.

Vertical displacement was computed by subtracting the vertical height of the object at the end of the lift from the height at the beginning. Reference displacements were calculated accordingly using the reference heights mentioned previously. The reference values for the six main source–destination pairs are shown in Table 3. The extra lifts from Table 2 were not processed by this algorithm, so the seven source–destination pairs unique to those lifts are not included in Table 3.

Like the vertical height estimations, the horizontal distance depends on the hand position measurements provided by the Xsens software. The horizontal distance is calculated as the distance in the floor plane from the midpoint between the participant’s feet to the object. Like asymmetry, the horizontal distance that the object was held from the body was not controlled during the experiment. Participants were allowed to hold the object at whatever distance felt comfortable to them. However, a tape measure was used to get an approximation of the horizontal distance for each of the sources/destinations. These reference distances are 0.3 m, 0.5 m, 0.45 m, and 0.45 m for the floor, knee, chest, and head levels, respectively.

### 2.5. Lift Classification

The classification problem was split into four subproblems: posture, twisting direction, vertical movement, and weight. The classes for each subproblem are defined as follows.

1. Posture = stooping, squatting, neither
2. Twisting = left, right, neither
3. Vertical Movement = floor to chest, chest to floor, knee to chest, chest to knee, chest to head, head to chest
4. Weight = 3 kg, 10 kg

To make these classifications, a pool of 223 features was extracted from the lifts. The joint movements along all 3 axes for 28 joints provided by the Xsens software were included. The joint movement is computed as $\theta_{f}-\theta_{s}$, where $\theta_{f}$ is the angle of the joint at the end of the lift and $\theta_{s}$ is the angle at the beginning. The magnitudes of these joint movements (i.e., their absolute values) were included as separate features, just in case they were more useful for some of the classification problems. The mean absolute velocities and accelerations of 23 body segments were also included. The remaining 9 features were the parameters estimated from the IMU data, described in Section 2.4. These included the height of the object above the floor, the horizontal distance from the lifter to the object, and the asymmetry angle for both the start and end of each lift. They also included the vertical displacement of the object, the largest-in-magnitude asymmetry angle, and the difference in asymmetry angle between the start and end of the lift. The extra lifts from Table 2 were not included in classification of vertical movement, because there were not enough samples to train the classifier on the unique vertical movement categories contained in the those lifts. Like the lift parameters, classifications were made for both manually and automatically labeled start/end times.

With 223 features, this was a high-dimensionality problem that needed to be reduced. Two approaches to dimensionality reduction were attempted: neighborhood component analysis (NCA) [30] and principal component analysis (PCA) [31]. We tried dimensionality reduction for each subproblem, with the exception of vertical movement, and found that the preliminary classification results were better with NCA, so that is what we used for the final feature selection. The starting and ending vertical heights of the object, 2 features already included in the feature pool, proved to be sufficient for the vertical movement subproblem, so automatic feature selection was not used. For the other subproblems, NCA assigned weights to all the features based on correlation with the classification categories. Features with the highest 2% (a relative threshold based on the highest weight in each subproblem) weights were tried in various combinations to find the best-performing feature vector for each subproblem. Then, some features were manually removed from these vectors because we suspected they were picking up on experimental patterns and artificially boosting the results. For example, one of the top-weighted features for the weight subproblem was the maximum twist angle of the participant’s upper body. If we consider that all low-weight lifts performed were straight lifts, it becomes clear that selecting this feature would immediately eliminate all asymmetric lifts; therefore, it was removed from the feature vector for the weight subproblem. Other features removed for the weight subproblem were the angle movement between the pelvis and the T8 vertebra along the Y axis (vertical while standing) and the horizontal start/end hand positions. The pelvis-T8 angle movement was removed for the same reason as the maximum twist angle, and the horizontal hand positions were removed because the main low-weight lifts only involved 2 vertical levels (chest and floor), which had fairly consistent horizontal distance measurements across all participants. We did not want the algorithm to classify weight based on the shelf levels used during the lift. Vertical start/end hand positions were removed from the feature vector for the posture subproblem, because the vertical position of the hands should not be indicators of whether the participant was stooping or squatting.

Multiple classifiers were trained on each subproblem to determine the best model for each. Among the models tested were decision trees, naive Bayes, linear discriminant analysis (LDA), support vector machine (SVM), and k-nearest neighbor (KNN). Uniform prior probabilities were assumed. Each classifier was verified with subject-independent 5-fold cross-validation. In subject-independent cross-validation, the folds are created in such a way that every participant’s trials appear in exactly 1 fold. This ensures that the test data were never from the same participant as the training data. Because there were 24 participants, 1 of the folds contained 4 participants instead of 5. The results from the best classifiers are reported in Section 3. For all subproblems, the best results were obtained with KNN classifiers. However, KNN was deemed an inappropriate classifier to use for the weight subproblem, as the ratio of 3 kg lifts to 10 kg lifts is very low (4:26) and the feature spaces overlap severely. If KNN were to be used, the classification would almost certainly be biased toward 10 kg. For this reason, a naive Bayes classifier was chosen for the weight subproblem.

## 3. Results

All of the lifting trials recorded (shown in Table 2) were used for parameter estimation and classification. There were no trials lost to data corruption, participant fatigue, etc. Raw data from all participants are available as Supplementary Materials.

### 3.1. Lift Detection

The lift detection algorithm estimated both the start and end times of each lift. The times reported are the number of seconds since the beginning of the recording. Error was calculated as $T_{est}-T_{actual}$, where $T_{est}$ is the estimated lift start/end time and $T_{actual}$ is the manually labeled start/end time. The error for start time estimates across all 1489 lift trials was 0.10 s ± 0.21 s. The error for end time was 0.36 s ± 0.27 s. To put this into perspective, the lift duration was 2.52 s ± 0.56 s and the trial duration was 7.74 s ± 1.09 s. The algorithm always detected a lift (i.e., there were no false negatives). This was expected, as it simply detects peaks in the distance from center of mass to hands during the trials.

### 3.2. Parameter Estimation

#### 3.2.1. Asymmetry

Table 4 shows the asymmetry measurement for each lift type (no twist, twist left, and twist right) with their respective errors.

The mean twist amount for the straight lifts is very close to 0. The means for the left and right asymmetric lifts are larger in magnitude than the expected values.

#### 3.2.2. Vertical Height and Displacement

The algorithm was used to estimate the vertical height of the object at the beginning and ending of every lift. Table 5 summarizes the vertical height measurements. The results of the vertical displacement estimation are shown in Table 6.

#### 3.2.3. Horizontal Distance

Table 7 shows the reference horizontal distance measurements compared to the measurements.

### 3.3. Lift Classification

Optimal features were selected for each classification subproblem, as described in Section 2.5. Seven features were selected for posture, three for asymmetry, three for vertical movement, and three for weight:

1. Posture: absolute angle between the vertical plane and the participant’s T8 vertebra, absolute joint movements of the left and right knees on the flexion–extension axis, absolute joint movements of the left and right shoulders on the flexion–extension axis, and absolute joint movements of the left and right hips on the flexion–extension axis.
2. Asymmetry: difference in twist angle from the beginning to the end of the lift, the largest twist angle during the lift, and the T9 to T8 joint movement along the X axis, which extends forward in the direction the person is facing.
3. Vertical Movement: vertical displacement of the object, average vertical starting position of the left and right hands, and the average vertical ending position of the left and right hands.
4. Weight: average absolute velocity of the left and right hands and the average absolute velocity of the head.

Table 8 summarizes the classification results for the four subproblems. Classification results for both automatically and manually labeled start/end times are included.

## 4. Discussion

### 4.1. Discussion of Results

#### 4.1.1. Lift Detection

**Figure 5:**
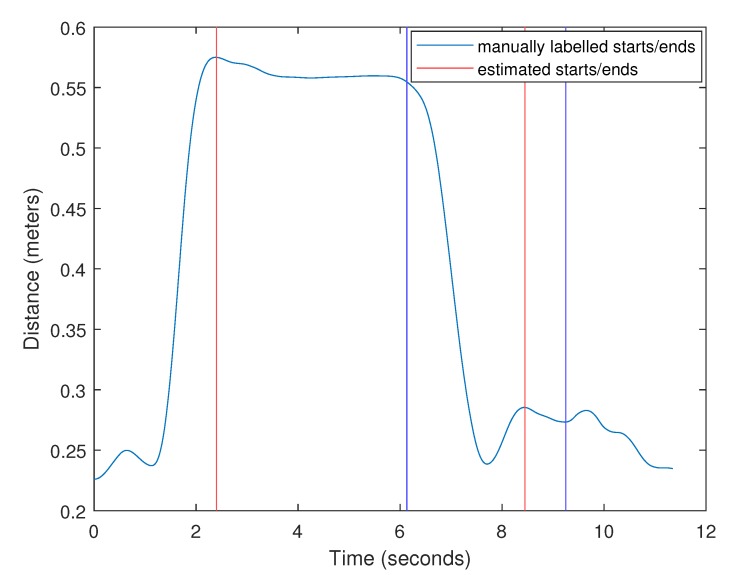
Lift start/end detection using the distance from the center of mass to the hands. The start time was mislabeled by the lift detection algorithm in this lift due to the participant reaching for the crate early.

The lift detection algorithm is accurate when participants do nothing but the lift during the recording. Spontaneous movements, such as waving a hand or reaching down to tie shoelaces, have the potential to cause false positives. In this experiment, the only cause of this was when participants over-anticipated the signal to begin the lift and began reaching for the object early, only to stop before grabbing it. In these lifts, the participants moved their arms toward the object early and held them there until instructed to begin the lift, creating a large plateau in the distance from center of mass to the hands. The algorithm does not have any way of identifying these false starts, so the beginnings of the lifts were occasionally mislabeled. In a few of the trials, this led to extremely large errors. The worst of these is shown in Figure 5. In this example, the beginning of the lift was detected at the false start and the lift did not actually begin until 3.68 s later. Because there was not a prominent local maximum at the actual start time, the algorithm was able to correctly identify the end time. For reference, the actual lift was only 3.12 s long.

#### 4.1.2. Parameter Estimation

Based on the asymmetry estimation results in Table 4, it appears that the algorithm has a tendency to overestimate twist angles slightly. This is likely due to not controlling the exact twist amount participants were required to use. When performing asymmetric lifts, hands have the ability to translate the object side-to-side, effectively increasing the twist range. Subjects may have tended to place the object closer to the center of the offset shelf during asymmetric lifts, which would have increased the asymmetry measurement. Overall, the twist angle measurements are very precise and consistent across all participants, which makes it a viable option for real-time characterization.

The vertical height estimates are very precise, as shown by the low standard deviations in Table 5. However, they lack accuracy at the extremes. It appears that the Xsens software tends to overestimate the height of the hands when a person is bending over, and underestimate when a person is reaching above their head. In the middle ranges (knee and chest), the vertical height estimates are quite accurate. This is possibly due to limitations in the Xsens musculoskeletal body model. The hand IMUs were placed on the backs of the participants’ hands, and they were able to rotate their wrists freely, which may have also contributed to some of the error.

Due to the tendencies of the algorithm to underestimate object positions at the extremes, the absolute displacement is generally lower than expected for low-to-high lifts and higher than expected for high-to-low lifts. The standard deviation of the absolute error is low, as shown in Table 6, which means the method is at least consistent.

There was significant variation in the horizontal distance measurements across trials. This was expected, as participants were able to hold the crate at any distance they desired. Because of this, it is impossible to determine how much of the error was due to lift variation and how much was due to the method and sensors.

Overall, the parameter estimation algorithms performed very similarly between the manually labeled and automatically labeled lifts. Of course, the cases where the lift detection algorithm mislabeled a start or end have very poor parameter estimation results. To counter this, it could be possible to use additional features, such as inertial coupling between the hands [24], as a redundant check to make sure that a lift is actually occurring between the detected start and end times. If one is not, the algorithm can strategically search for better times.

#### 4.1.3. Lift Classification

The classification accuracies from Table 8 for the main three subproblems (posture, asymmetry, and vertical movement) are quite high. By comparison, the weight classifier performed poorly. Part of the problem could be that there were not enough samples of low-weight lifts in the training data. The biggest issue, however, was most likely the fact that distinguishing between 3 and 10 kg weights with only IMUs is very difficult and there are probably no accurate features for classification. Because so few low-weight lifts were performed, and they were the same lifts for each participant, it is very easy for a classification algorithm to overfit to the training data. We attempted to fix this by removing “bad” features from the automatically selected features, but it is possible that the algorithm still shows some bias. Better features could exist that were not in the feature pool, but we chose not to pursue it further for this study because it was not the main goal, and we did not believe we would be able to reliably distinguish 3 kg from 10 kg using only motion data. Another possibility of improving classifier performance across all subproblems is to use ensemble classifiers, which combine the results from multiple classification algorithms to produce 1 (potentially better) classification [32].

It is worth mentioning that our method is subject-nonspecific. That is, the classification algorithms are trained on potentially different participants than they are used on. This is desirable in many cases, because it eliminates the need for training data to be collected from each individual participant before use, and therefore makes the product more widely marketable. However, subject-specific classifiers can often obtain better results with less data than their subject-nonspecific counterparts [26].

### 4.2. Conversion to Real-Time

A major limitation of the methods developed in this study is that they depend on presegmentation of the trials, which would not be possible in real-time applications. For lift detection, we assumed that there was only 1 lift per trial and that the hands always started from the same position (relaxed, at the participants’ sides). For cases where multiple lifts are performed back-to-back (i.e., person lifts box from floor to shelf and then back to floor), modifications would have to be made to ensure both lifts are detected. The biggest challenge in developing the algorithms for real-time would be identifying the start and end of a lift amidst a continuous stream of data. Lu et al. demonstrated one plausible method for accomplishing this using a sliding pattern recognition window [24]. Once that problem is solved, the analysis could be done the same way it is done offline. This would work fine for health monitoring applications, where lifting data are collected throughout the day and compiled into a report, but not for guiding assistive devices, which need classification results before the lift is finished so they can assist the user properly. For these applications, classification will need to be started as soon as possible and updated periodically throughout the lift. Posture, for example, could be classified in real-time by checking each individual frame for the conditions of stooping and squatting and reporting the most likely category along with a confidence value. This would allow an assistive device to decide which action it should take to help the user with the lift.

### 4.3. Applications

Lift characterization using IMUs has many potential applications. It could be useful for health monitoring, where lifters’ behaviors are analyzed over an extended period of time to obtain statistics about the types of lifts they perform. In a factory setting, this could be used to engineer safer processes and to help train workers on the correct procedures [2]. It could also be used to help patients suffering from musculoskeletal disorders recover or avoid further injury, especially if their occupations require frequent manual lifting. If a real-time algorithm was developed, the lift characterization data could be used to control assistive devices. For instance, an exoskeleton could be provided with lift information in order to properly support the user during lifting tasks, thereby increasing the effectiveness of such a device [3]. Such intent-detecting controllers have been designed for assistive devices before, such Parri et al.’s whole-body awareness controller for an active transfemoral prosthesis [13]. Their system uses lower limb kinematics computed using inertial sensors to recognize eight different behaviors, including quiet standing, quiet sitting, step-by-step stair ascent, walking (all the gait phases), sitting down, standing up, initiating walking, and terminating walking. The state of the user is decoded from the sensor signals, then used in a finite-state machine to drive the device actuators and make the task easier (or possible). Currently, the controller does not recognize lifting activity, so it is possible that real-time versions of the lifting algorithms developed in this study could be implemented in the future. The independent phases of the lift (e.g., grasping, releasing, entering squat, and standing back up from squat) could be detected and used to control the actuators accordingly. This would open up many new possibilities for active assistive devices.

As the IMU motion tracking system and presented algorithms were calibrated/trained only to healthy young adults, recalibration/retraining may be necessary for other participant samples. For instance, the posture and hand positions of older individuals that exhibit stooped posture may be inaccurately measured by these methods. This limits the use of these algorithms in general applications, where users may vary greatly in physical capability. Future work could focus on generalizing these algorithms for practical applications.

In this study, we assumed that both hands are used to perform a lift. However, many lifts can be performed with a single hand, such as picking up a kettlebell, as was done by Brandt et al. [23]. Most of the presented algorithms would not work properly for single-handed lifts, as most of them depend on the mean of the positions of the hands. This limits the applications of the methods in their current form, but minor modifications could be made to automatically detect which hand is being used for the lift and only take that hand into account.

## 5. Conclusions

This paper builds on previous studies by demonstrating the viability of lift detection and characterization using only wearable inertial sensors. With data pre-segmented into individual lifting trials, lift detection can be achieved using an algorithm which utilizes the estimated distance from the participant’s hands to his center of mass. Our algorithm had a start time error of 0.10 s ± 0.21 s and an end time error of 0.36 s ± 0.27 s. Once the beginning and ending of lifts have been identified, estimations of lifting parameters, such as vertical displacement, vertical starting and ending positions, horizontal distance, and asymmetry angle, can all be computed. Classification algorithms can also be used to classify the posture, twist direction, and vertical movement with very high accuracy, while weight of the object is quite difficult to classify using only IMUs. The classifiers in this study achieved accuracies of 96.78%, 98.32%, 97.28%, and 64.21% for posture, asymmetry, vertical movement, and weight, respectively, with automatic lift detection.

Although these algorithms were developed for offline lift classification, there is potential to expand them to online in the future so that they may be used for applications such as real-time health monitoring and assistive device control. The biggest obstacle to overcome in this regard is detecting the beginning and ending of lifts in real-time. Therefore, further work must be done in real-time detection of lifts using wearable inertial sensors.

## Figures and Tables

**Table 1:** The body positions of the Xsens IMU trackers.

Location	Position
Feet	Middle of bridge of both feet
Lower legs	Flat on the shin bones (medial surface of the tibia)
Upper legs	Lateral sides above knees
Pelvis	Flat on sacrum
Sternum	Flat, in the middle of the chest
Shoulders	On the Scapula (shoulder blades)
Upper arms	Lateral sides above elbows
Forearms	Lateral and flat side of the wrists
Hands	Backsides of both hands
Head	On the back of the head (held on with headband)

**Table 2:** All combinations of source, destination, posture, and twist direction that were used in the experiment, along with the number of each that were planned and that actually occurred throughout all 24 experiments. The table is organized by main lifts (the 30 main lifts that we were interested in), extra lifts (lifts designed specifically to serve as transition lifts), and unplanned lifts (lifts that were performed by mistake but do not necessarily hinder results). Though we required 48 occurrences of each of the main lifts (2 of each per participant), some were planned more times as transition lifts for some participants. The extra lifts were only performed when necessary as a transition between 2 main lifts.

Main Lifts							
No.	Source	Destination	Posture	Twist Direction	Weight (kg)	Planned	Occurred
1	floor	chest	squatting	straight	10	51	51
2	floor	chest	stooping	straight	10	50	48
3	knee	chest	squatting	straight	10	51	48
4	knee	chest	stooping	straight	10	50	50
5	chest	head	–	straight	10	48	46
6	floor	chest	squatting	straight	3	48	48
7	floor	chest	stooping	straight	3	48	50
8	chest	floor	squatting	straight	10	48	48
9	chest	floor	stooping	straight	10	51	48
10	chest	knee	squatting	straight	10	50	48
11	chest	knee	stooping	straight	10	49	49
12	head	chest	–	straight	10	48	46
13	chest	floor	squatting	straight	3	48	48
14	chest	floor	stooping	straight	3	48	50
15	floor	chest	squatting	left	10	49	49
16	floor	chest	stooping	left	10	48	48
17	floor	chest	squatting	right	10	48	47
18	floor	chest	stooping	right	10	48	48
19	knee	chest	squatting	left	10	49	51
20	knee	chest	stooping	left	10	48	48
21	knee	chest	squatting	right	10	48	48
22	knee	chest	stooping	right	10	49	49
23	chest	floor	squatting	left	10	48	48
24	chest	floor	stooping	left	10	48	48
25	chest	floor	squatting	right	10	48	48
26	chest	floor	stooping	right	10	48	48
27	chest	knee	squatting	left	10	48	48
28	chest	knee	stooping	left	10	48	47
29	chest	knee	squatting	right	10	48	50
30	chest	knee	stooping	right	10	48	48
**Extra Lifts**							
31	knee	floor	squatting	straight	10	1	1
32	knee	floor	stooping	straight	10	1	1
33	floor	head	squatting	straight	10	1	1
34	floor	knee	stooping	straight	10	4	4
35	head	knee	–	straight	10	1	1
36	chest	chest	–	left	10	4	4
37	chest	chest	–	right	10	9	9
38	floor	knee	squatting	left	10	2	2
39	floor	knee	squatting	right	10	1	1
40	knee	floor	stooping	left	10	1	1
41	knee	knee	squatting	left	10	1	1
42	floor	floor	squatting	left	10	1	1
43	floor	floor	stooping	left	10	1	1
44	floor	knee	stooping	right	10	1	1
45	knee	knee	stooping	right	10	1	1
**Unplanned Lifts**							
46	head	chest	–	straight	3	0	2
47	chest	head	–	straight	3	0	2
48	knee	chest	squatting	straight	3	0	2
49	chest	knee	squatting	straight	3	0	2

**Table 3:** The vertical displacement reference values in meters.

Floor to Chest	Chest to Floor	Knee to Chest	Chest to Knee	Chest to Head	Head to Chest
1.08	−1.08	0.54	−0.54	0.49	−0.49

**Table 4:** The asymmetry angles and errors measured across all 1489 lifts, separated by twist direction. Twists to the left (counterclockwise when viewed from above) are positive angles, while twists to the right are negative. Error was calculated as the estimated angle minus the reference angle. All values are reported in degrees.

		Manually Labelled		Automatically Labelled	
Twist Direction	Expected	Measured	Mean Error	Measured	Mean Error
no twist	0	−0.4±14.1	−0.4	−0.7±15.3	−0.7
twist left	45	51.8±11.8	6.8	51.9±11.2	6.9
twist right	−45	−51.7±11.7	−6.7	−51.8±12.0	−6.8

**Table 5:** The vertical height of the object was estimated for the beginning and ending of every lift. The error was calculated as the estimated height minus the reference height. The measured values and error are shown for each of the 4 possible vertical levels. All values shown are in meters.

		Manually Labelled		Automatically Labelled	
Level	Expected	Measured	Mean Error	Measured	Mean Error
floor	0.300	0.452±0.066	0.152	0.514±0.089	0.214
knee	0.840	0.845±0.049	0.005	0.861±0.053	0.021
chest	1.380	1.283±0.043	−0.097	1.286±0.045	−0.094
head	1.870	1.641±0.056	−0.229	1.640±0.055	−0.230

**Table 6:** The vertical displacement estimates and errors for each lift type. The vertical displacement error is calculated as $v_{est}-v_{actual}$, where $v_{est}$ is the estimated vertical displacement and $v_{actual}$ is the actual vertical displacement. Distances are displayed in meters.

		Manually Labelled		Automatically Labelled	
Vertical Movement	Expected	Measured	Mean Error	Measured	Mean Error
floor to chest	1.08	0.832±0.070	−0.248	0.796±0.077	−0.284
chest to floor	−1.08	−0.836±0.074	0.244	−0.752±0.109	0.328
knee to chest	0.54	0.432±0.041	−0.109	0.433±0.042	−0.107
chest to knee	−0.54	−0.451±0.041	0.089	−0.426±0.047	0.114
chest to head	0.49	0.384±0.026	−0.106	0.379±0.027	−0.111
head to chest	−0.49	−0.395±0.043	0.095	−0.404±0.062	0.086

**Table 7:** The horizontal distance between the participant and the object was estimated at the beginning and ending of every lift. Error is calculated as $h_{est}-h_{actual}$, where $h_{est}$ is the estimated horizontal distance and $h_{actual}$ is the reference horizontal distance. Distances are displayed in meters.

		Manually Labelled		Automatically Labelled	
Level	Expected	Measured	Mean Error	Measured	Mean Error
floor	0.300	0.248±0.081	−0.052	0.244±0.078	−0.056
knee	0.500	0.500±0.078	0.000	0.483±0.077	−0.017
chest	0.450	0.592±0.094	0.142	0.575±0.087	0.125
head	0.450	0.559±0.087	0.109	0.554±0.074	0.104

**Table 8:** Classification results for the 4 subproblems: posture, asymmetry, vertical movement, and weight. As the feature extraction process depended on the estimated start/end times, classification results for both automatically and manually labeled start/end times are included.

Subproblem	Number of Classes	Accuracy Manually Labelled	Accuracy Auto Labelled	Classification Method
Posture	3	97.18%	96.78%	KNN, k = 30
Asymmetry	3	98.27%	98.32%	KNN, k = 10
Vertical Movement	6	99.60%	97.28%	KNN, k = 10
Weight	2	65.74%	64.21%	Naive Bayes

## Supplementary Materials

The IMU data collected for this study are available online at http://doi.org/10.5281/zenodo.3724998.

## Abbreviations

The following abbreviations are used in this manuscript:

GPS	Global positioning system
IMU	Inertial measurement unit
KNN	K-nearest neighbor
LDA	Linear discriminant analysis
NCA	Neighborhood component analysis
NIOSH	National Institute for Occupational Safety and Health
PCA	Principal component analysis
RNLE	Revised NIOSH lifting equation
sEMG	Surface electromyography
SVM	Support vector machine
